# Kinematic Analysis of Healthy Hips during Weight-Bearing Activities by 3D-to-2D Model-to-Image Registration Technique

**DOI:** 10.1155/2014/457573

**Published:** 2014-11-23

**Authors:** Daisuke Hara, Yasuharu Nakashima, Satoshi Hamai, Hidehiko Higaki, Satoru Ikebe, Takeshi Shimoto, Masanobu Hirata, Masayuki Kanazawa, Yusuke Kohno, Yukihide Iwamoto

**Affiliations:** ^1^Department of Orthopaedic Surgery, Graduate School of Medical Sciences, Kyushu University, 3-1-1 Maidashi, Higashi-ku, Fukuoka 812-8582, Japan; ^2^Department of Medical Engineering, Faculty of Engineering, Kyushu Sangyo University, 2-3-1 Matsugadai, Higashi-ku, Fukuoka 813-0004, Japan; ^3^Department of Mechanical Engineering, Faculty of Engineering, Fukuoka Institute of Technology, 3-30-1 Wajiro-higashi, Higashi-ku, Fukuoka 811-0295, Japan

## Abstract

Dynamic hip kinematics during weight-bearing activities were analyzed for six healthy subjects. Continuous X-ray images of gait, chair-rising, squatting, and twisting were taken using a flat panel X-ray detector. Digitally reconstructed radiographic images were used for 3D-to-2D model-to-image registration technique. The root-mean-square errors associated with tracking the pelvis and femur were less than 0.3 mm and 0.3° for translations and rotations. For gait, chair-rising, and squatting, the maximum hip flexion angles averaged 29.6°, 81.3°, and 102.4°, respectively. The pelvis was tilted anteriorly around 4.4° on average during full gait cycle. For chair-rising and squatting, the maximum absolute value of anterior/posterior pelvic tilt averaged 12.4°/11.7° and 10.7°/10.8°, respectively. Hip flexion peaked on the way of movement due to further anterior pelvic tilt during both chair-rising and squatting. For twisting, the maximum absolute value of hip internal/external rotation averaged 29.2°/30.7°. This study revealed activity dependent kinematics of healthy hip joints with coordinated pelvic and femoral dynamic movements. Kinematics' data during activities of daily living may provide important insight as to the evaluating kinematics of pathological and reconstructed hips.

## 1. Introduction

The hip joint achieves great mobility and stability during various activities of daily living. Participation in specific activities requires a complex range of hip movements and muscle activity. Video motion capture system with reflective markers has been widely used for* in vivo* joint kinematic analysis [1–4]. However, external markers attached to the skin could be affected by soft tissue artifacts with substantial errors [5–7]. Previous studies have reported direct measurement of skeletal kinematics from 3D surface models and radiographic image sequences [8–13]. However, no studies have employed 3D-to-2D model-to-image registration technique to analyze* in vivo* healthy hip kinematics. Kinematic analysis of healthy hips during functional weight-bearing activities is one key to evaluating kinematics of pathological and reconstructed hips. In particular, understanding dynamic 3D kinematics of deep flexion and rotation could enhance the opportunity to identify abnormal hip kinematics.

The purposes of this study were to evaluate kinematics of healthy hips during gait, squatting, chair-rising, and twisting by 3D-to-2D model-to-image registration technique. Each activity is one of the fundamental and commonly encountered activities in daily living, including deep flexion and rotation. Specific questions to be addressed include the following. (1) Is the accuracy of the measurement technique sufficient to assess hip kinematics? (2) How much are flexion/extension and axial rotation of pelvis, femur, and hip joints typical of gait, chair-rising, squatting, and twisting activities? (3) How much different is the kinematics of squatting from chair-rising, the same type of hip flexion activity?

## 2. Materials and Methods

This study consisted of six healthy male subjects, averaging 33 years (31–36), 173 cm (170–177), and 67 kg (56–80). No subjects had experienced any hip injury or surgery or had any abnormity in radiographic images of bilateral hip joints. All subjects were given informed consent to participate in this Institutional Review Board approved study (IRB number 24–55) and were informed of the risk of radiation exposure required. Data were handled in accordance with the Ethical Standards of the Helsinki Declaration.

**Figure 1:**
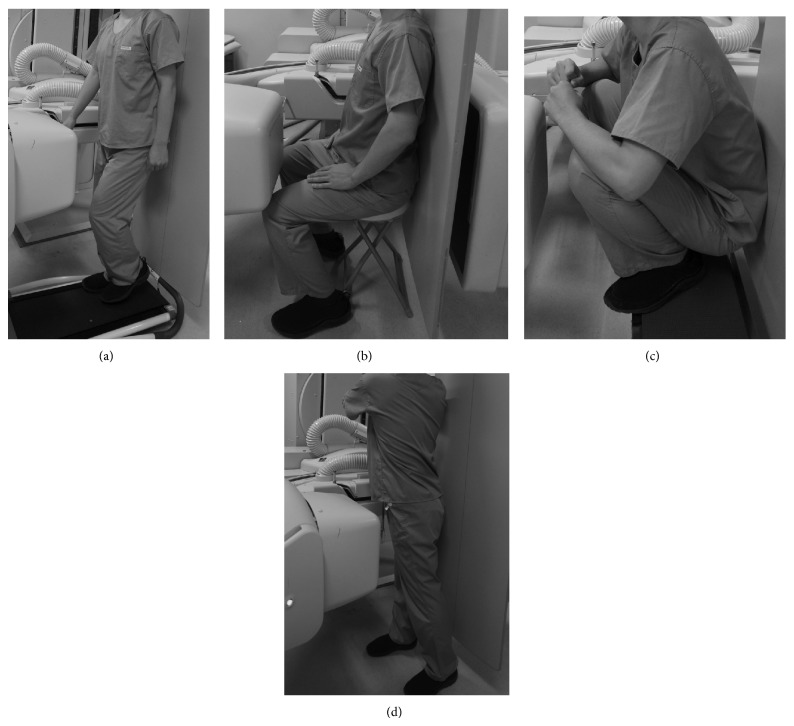
Subjects walked on a level treadmill at 1.0 km/hour (a), got up from a chair (b), stood up from the maximum hip flexed position (c), and rotated the trunk bilaterally from a neutral standing position (d).

**Figure 2:**
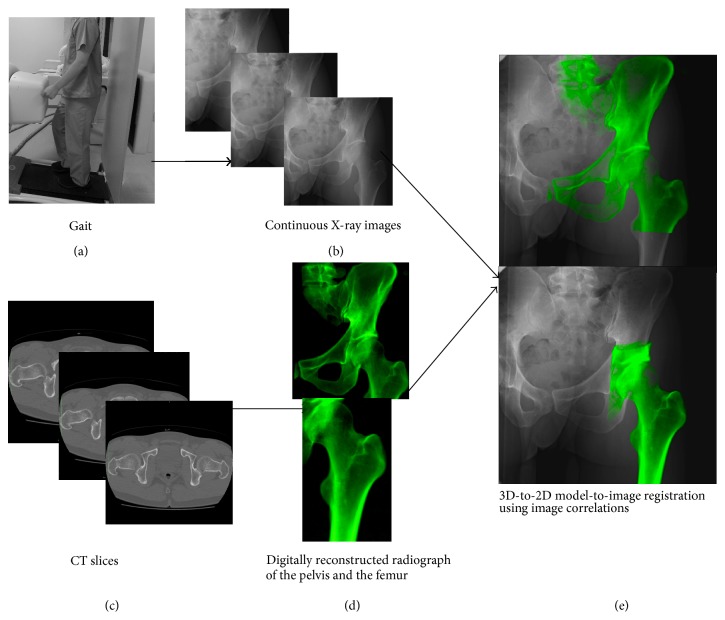
The hip motions (a) were captured as continuous X-ray images using a flat panel X-ray detector (b). CT slices (c) were reconstructed to the density-based digitally reconstructed radiograph (d) and projected onto radiographic images (e). The 6 degrees of freedom of the pelvis and femur were determined by 3D-to-2D model-to-image registration technique using image correlations.

Continuous anteroposterior radiographic images of gait, chair-rising, squatting, and twisting motions were recorded using a flat panel X-ray detector (Ultimax-I, Toshiba, Tochigi, Japan) with an image area of 420 mm (H) × 420 mm (V) and 0.274 mm × 0.274 mm/pixel resolution. The frame rate was set at 3.5 frames/sec to acquire high-resolution images. For gait, subjects walked on a level treadmill at 1.0 km/hour (Figures 1(a), 2(a), and 2(b)). For chair-rising and squatting, subjects got up from sitting position on a chair with 46.5 cm height (Figure 1(b)) and maximum hip flexed position (Figure 1(c)). For twisting, subjects rotated their trunk contralaterally and ipsilaterally from standing position while keeping their feet on the floor (Figure 1(d)).

The 3D positions and orientations of the pelvis and femur in movement cycle were determined by 3D-to-2D model-to-image registration technique using image correlations (Figure 2). The relative geometric relationship between the X-ray source and the projection plane of the flat panel X-ray detector system was determined using a coordinate building frame [13, 14]. Each subject was scanned by computed tomography (CT; Aquilion, Toshiba, Tochigi, Japan) with a 512 × 512 image matrix, 0.35 × 0.35 pixel dim, and 1 mm thickness spanning from superior edge of the pelvis to below the knee joint line (Figure 2(c)). The 3D digital image was constructed in a virtual 3D space by the CT data, and computer simulation of the radiographic process was carried out to generate virtual digitally reconstructed radiograph (DRR), in which the light source and projection plane parameters were set identical to the actual flat panel X-ray detector imaging conditions [13] (Figure 2(d)). The density-based DRRs were then compared with the serial X-ray images acquired using the flat panel X-ray detector (Figure 2(e)). Correlations of the pixel values between the DRRs and real X-ray images were used to fine-tune the 3D model. In practical terms, the simulation images were constructed by repositioning the 3D digital image in 6 degrees of freedom, and the matching fitness was by counting the voxels that did not correspond between the constructed images and a flat panel X-ray detector derived X-ray scans (i.e., exclusive disjunctions).

**Figure 3:**
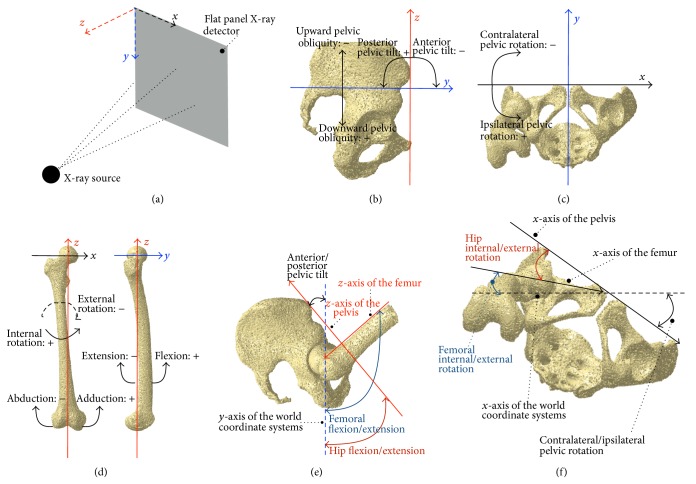
The world coordinate systems and the coordinate systems of the pelvis and femur were based on the projected plane of a flat panel X-ray detector (a), the anterior pelvic plane ((b) and (c)), and the center of the femoral head and the transepicondylar axis (d), respectively. The relative positions and orientations of the pelvis and femur for the world coordinate systems were defined as pelvic and femoral movements, respectively. The relative femoral positions and orientations for the pelvis were defined as hip movements ((e) and (f)).

The upper left end point on the projection plane of a flat panel X-ray detector was defined as the world coordinate system origin [13, 14] (Figure 3(a)). The mediolateral (*x*-) and superoinferior (*y*-) axes were horizontal and perpendicular to the floor, respectively. The anteroposterior (*z*-) axis was formed from the cross product of the first two. Anatomical coordinate systems of the pelvis and femur were embedded in each density-based volumetric bone model. The midpoint of the bilateral anterior superior iliac spine (ASIS) was defined as the coordinate system origin for the pelvis (Figures 3(b) and 3(c)). The mediolateral (*x*-) axis of the pelvis was defined by a line passing through the bilateral ASIS. The proximal/distal (*z*-) axis of the pelvis was defined by a line perpendicular to* x*-axis in the anterior pelvic plane (APP). The anteroposterior (*y*-) axis was formed from the cross product of the first two. The center of the femoral head was defined as the coordinate system origin for the femur (Figure 3(d)). The mediolateral (*x*-) axis of the femur was defined by a line parallel to the transepicondylar axis (TEA) in the plane intersecting the origin. The proximal/distal (*z*-) axis of the femur was defined by a line perpendicular to the* x*-axis in the plane intersecting the origin and the midpoint of TEA. The anteroposterior (*y*-) axis was formed from the cross product of the first two.

The relative positions and orientations of the pelvis with respect to the world coordinate systems were defined as pelvic movements (anterior/posterior tilt, upward/downward obliquity, and contralateral/ipsilateral rotation; Figures 3(b) and 3(c)), and those of the femur with respect to the world coordinate systems were defined as femoral movements (flexion/extension, adduction/abduction, and internal/external rotation; Figure 3(d)). We also defined the relative positions and orientations of the femur for the pelvis as hip movements (flexion/extension, adduction/abduction, and internal/external rotation; Figures 3(e) and 3(f)).

An accuracy evaluation experiment was performed on a pelvis and femur of a pig carcass [11, 13]. The pelvis and femur fixed to a stage were rotated and translated to known values [13, 14]. For each position, three X-ray scans were taken, and the 3D-to-2D model-to-image registration technique was performed for the radiographic images at each position to determine the orientations and positions of each bone. The measurement accuracy was evaluated using the root-mean-square (RMS) errors.

Values were expressed as the mean ± standard deviation. Repeated measures analysis of variance and post hoc tests (paired* t*-test) were used to compare chair-rising and squatting by JMP Software (Version 10.0; SAS Institute, Cary, NC). Probability values < 0.05 were considered significant.

## 3. Results and Discussion

The accuracy evaluation experiment demonstrated that the RMS errors of the pelvis and femur were 0.21 mm and 0.15 mm in the in-plane direction, 0.14 mm and 0.23 mm in the out-of-plane direction, and 0.25° and 0.23° in rotation, respectively. Recently, the feasibility of the 3D-to-2D model-to-image registration techniques was assessed for kinematic analyses of cadaveric hip joints [15, 16]. Martin et al. reported that the precision measurements of the DRRs and biplane X-ray images averaged 0.3 mm for translational variables and 0.8° for rotational variables [15]. Also, Lin et al. reported that the repeatability of the dual fluoroscopic imaging system technique was less than ±0.77 mm and ±0.64° in position and orientation for measuring hip kinematics [16]. The RMS errors in this study were equivalent to the results of the previous studies using biplane radiography [15, 16]. This study first evaluated* in vivo* hip joint kinematics during daily life activities using density-based DRRs and flat panel X-ray detector.

**Figure 4:**
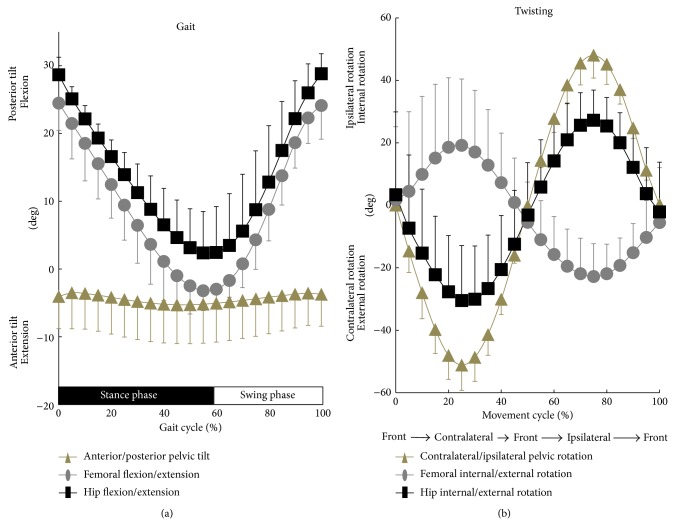
Anterior/posterior pelvic tilt and femoral and hip flexion/extension angles during gait (a). Contralateral/ipsilateral pelvic rotation and femoral and hip internal/external rotation angles during twisting (b).

For gait, the maximum/minimum anterior pelvic tilt angle was 6.0 ± 5.0°/2.7 ± 5.4° (Figure 4(a)). Subjects tended to tilt anteriorly around 4.4° on average during full movement cycle. Therefore, hip flexion angle was larger than femoral flexion angle throughout gait cycle. Although natural variability exists, our data generally agree with the literature available. Several studies have investigated hip kinematics during walking using motion capture system and showed anterior pelvic tilt in healthy young adults [1, 3, 7]. In this study, the maximum/minimum femoral and hip flexion angles were 25.7 ± 3.5°/−4.2 ± 2.8° and 29.6 ± 2.7°/1.3 ± 7.4°, respectively (Figure 4(a)). The hip adduction/abduction and internal/external rotation angles at the maximum/minimum hip flexion during gait were 0.9 ± 3.1°/−2.5 ± 2.1° of adduction and 2.3 ± 7.8°/0.2 ± 5.8° of internal rotation, respectively (Table 1). The femur demonstrated 4.2° of extension relative to the world coordinate system, but the healthy subjects do not necessarily stretch their hip joints into hyperextension during gait. Previous gait analyses have demonstrated approximately 10° of hip hyperextension at the terminal stance phase of gait [1, 3, 7]. The reason for the discrepancy could be explained by different measurement methods (video-based versus radiographic-based kinematic analyses), anatomic coordinate systems (skin markers-derived versus CT models-derived coordinate systems), gait speed (at 3.3–5.7 km/hour versus 1.0 km/hour), and conditions (on level ground versus treadmill).

**Figure 5:**
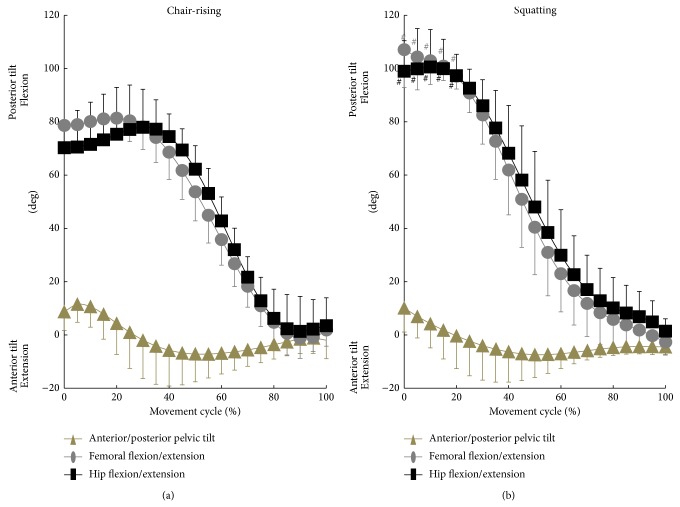
Anterior/posterior pelvic tilt and femoral and hip flexion/extension angles during chair-rising (a) and squatting (b). ^#^ Significantly different between chair-rising and squatting (*P* < 0.05).

For chair-rising and squatting, the maximum absolute values of anterior/posterior pelvic tilt were 12.4 ± 7.3°/11.7 ± 9.4° and 10.7 ± 8.1°/10.8 ± 8.1°, respectively (Figure 5). The pelvis began to tilt anteriorly from posterior tilt around 10% and 0% of the movement cycle, respectively. Pelvis tilted most anteriorly around 55% and 50% of the chair-rising and squatting cycles, respectively. Due to the posterior pelvic tilt, the maximum hip flexion angles during chair-rising and squatting (81.3 ± 13.6° and 102.4 ± 12.3°, resp.) demonstrated smaller angles than the maximum femoral flexion angles (83.5 ± 8.0° and 108.5 ± 13.1°, resp.; Table 1). There were no significant differences (*P* = 0.29 and 0.17, resp.) in the hip adduction/abduction and internal/external rotation angles at the maximum hip flexion during chair-rising (0.7 ± 5.9° of abduction and 22.5 ± 12.1° of external rotation) and squatting (7.0 ± 12.5° of abduction and 31.6 ± 8.7° of external rotation; Table 1). Few studies have analyzed healthy hip kinematics during squatting including deep flexion. Hemmerich et al. reported kinematics of healthy subjects during squatting using electromagnetic tracking system and demonstrated that the maximum hip flexion angles reached up to 95 ± 27° for squatting [17]. Lamontagne et al. examined pelvic motion without insight into neutral pelvic orientation and demonstrated that the change of pelvic tilt averaged 24 ± 7° during maximum squat [18]. The amount of anterior/posterior pelvic tilt and hip flexion angles in our study were consistent with the results of the previous studies [17, 18]. In this study, hip flexion during chair-rising and squatting peaked on 30% and 10% of movement because of the change of anterior/posterior pelvic tilt. Ganz et al. showed that hip pain in patients with femoroacetabular impingement often occurred on the way of movement [19]. Thus, evaluation of dynamic hip kinematics is important to diagnose and treat pathological hip conditions. We also found that there were no significant differences (*P* = 0.62) in anterior/posterior pelvic tilt between chair-rising and squatting (Figure 5). Both femoral and hip flexion/extension angles during squatting are significantly (*P* < 0.01) larger than those during chair-rising from 0% to 20% of movement cycle. These results indicate that sagittal plane pelvic tilt may not highly contribute to approximately 20° of further hip flexion in squatting compared to chair-rising contralateral/ipsilateral.

For twisting, the maximum absolute values of pelvic contralateral/ipsilateral and hip internal/external rotations were 48.0 ± 7.3°/51.1 ± 8.1° and 29.2 ± 13.5°/30.7 ± 17.3°, respectively (Figure 4(b) and Table 2). The hip rotation demonstrated the smaller absolute angles with respect to the pelvic rotation. Subjects tended to flex and abduct their hips during ipsilateral twisting (18.0 ± 13.7° of flexion and 6.4 ± 4.9° of abduction; Table 2). McGinley et al. previously reported that the highest error in kinematic measurements using skin markers-based motion capture was clearly found in hip rotation [7]. Therefore, few researchers have analyzed kinematics of torsional movements that are frequently required during daily living [20] and sports activities [21]. Wada et al. examined hip kinematics of healthy subjects during body rotation using skin markers and showed that the maximum angles of pelvic and hip internal/external rotations were 57.8° and 16.7°, respectively [4]. Our study demonstrated smaller amount of pelvic internal/external rotation but larger amount of hip internal/external rotation during twisting compared to the previous study [4]. In this study, physiological bilateral twisting required a large range of hip axial rotation, approximately 60°. These kinematic data should be beneficial for orthopaedic surgeons and primary care physicians to counsel patients with hip osteoarthritis or total hip prostheses regarding torsional activities.

This study has several limitations. First, the study included only young male hips, which could not represent the whole population. The pelvis of older subjects tended to tilt posteriorly with degenerative changes in the spine [1, 3], and sex differences have been found in temporal gait parameters [2]. Therefore, further kinematic study has to be done on the effects of aging and sex on the 3D hip kinematics. The number of subjects is similar to previous fluoroscopic studies that have analyzed four or five healthy joints [12, 22, 23] and is consistent with minimizing X-ray exposure to healthy individuals while still obtaining important information. Second, the current approach ignores articular cartilage and acetabular labrum, which are invisible on X-ray but obviously affect contact pattern. There is currently no X-ray-based technique that will overcome this limitation, but 3D-to-2D registration techniques have the ability to reveal continuous dynamic* in vivo* kinematics. Finally, sequential movements in squatting were collected twice because the flat panel X-ray detector still provided a limited field of view. However, our method could examine a variety of weight-bearing activities with sufficient accuracy.

## 4. Conclusions

Healthy hip kinematics were evaluated in four different functional weight-bearing activities over the range of 100° of flexion and 60° of axial rotation by 3D-to-2D model-to-image registration techniques. Accuracy of less than 0.3 mm in translation and 0.3° in rotation was equivalent to the results of the previous studies. This study revealed that healthy hip joints showed activity dependent kinematics with coordinated pelvic and femoral dynamic movements. Kinematic data in this study could be referred to as normative patterns of movement. Because pathological changes may influence hip kinematics, we currently evaluate patients with hip diseases including osteoarthritis and femoroacetabular impingement using this technique.

## Figures and Tables

**Table 1:** The hip adduction/abduction [adduction +, abduction −] and internal/external rotation [internal +, external −] angles at the maximum hip flexion during gait, chair-rising, and squatting.

Activities	Maximum hip flexion (°)	Hip adduction/abduction (°)	Hip internal/external rotation (°)
Gait	29.6 ± 2.7	0.9 ± 3.1	2.3 ± 7.8
Chair-rising	81.3 ± 13.6	−0.7 ± 5.9	−22.5 ± 12.1
Squatting	102.4 ± 12.3	−7.0 ± 12.5	−31.6 ± 8.7

Values are expressed as mean ± SD.

**Table 2:** The hip flexion/extension [flexion +, extension −] and adduction/abduction [adduction +, abduction −] angles at the maximum hip internal and external rotations [internal +, external −] during twisting.

Twisting	Hip flexion/extension (°)	Hip adduction/abduction (°)	Maximum hip internal and external rotations (°)
Contralateral	0.5 ± 5.7	−0.9 ± 3.5	−30.7 ± 17.3
Ipsilateral	18.0 ± 13.7	−6.4 ± 4.9	29.2 ± 13.5

Values are expressed as mean ± SD.
